# Ruthenium(II) complexes of curcumin and β-diketone derivatives: effect of structural modifications on their cytotoxicity

**DOI:** 10.1098/rsos.240353

**Published:** 2024-07-31

**Authors:** Flávia E. Jacinto, Letícia Pires de Oliveira, Alzir A. Batista, Katia M. Oliveira, Rodrigo S. Correa

**Affiliations:** ^1^ Department of Chemistry, Institute of Biological and Exact Sciences, Campus Morro do Cruzeiro, Federal University of Ouro Preto (UFOP), Ouro Preto, MG 35400-000, Brazil; ^2^ Department of Chemistry, Federal University of São Carlos (UFSCar), CP 676, São Carlos, SP 13561-901, Brazil; ^3^ Institute of Chemistry, University of Brasília (UnB) – Campus Darcy Ribeiro, Brasília, DF 70910-900, Brazil

**Keywords:** ruthenium(II) complexes, β-diketones, curcumin, antitumour, DNA, metallodrug

## Abstract

Ruthenium(II) complexes (**Ru1**–**Ru3**) with the general formula [Ru(O-O)(PPh_3_)_2_(bipy)]PF_6,_ bearing two triphenylphosphine (PPh_3_), bipyridine (bipy) and a series of natural and synthetic β-diketones (O,O) ligands were synthesized and characterized using various analytical techniques. The interaction between the complexes and *calf thymus* DNA (CT-DNA) was investigated and demonstrated a weak interaction. The cytotoxicity of the complexes was investigated against breast cancer cells (MDA-MB-231 and MCF-7), lung cancer cells (A549), cisplatin-resistant ovarian cancer cells (A2780*cis*), as well as non-tumour lung (MRC-5) and non-tumour breast (MCF-10A) cell lines. All complexes exhibited cytotoxic activity against all the cell lines studied, with half maximal inhibitory concentration (IC_50_) values ranging from 0.39 to 13 µM. Notably, the three complexes demonstrated selectivity against the A2780*cis* cell line, with IC_50_ ranging from 0.39 to 0.82 µM. Among them, Ru2 exhibited the highest cytotoxicity, with an IC_50_ value of 0.39 µM. Consequently, this new class of complexes shows good selectivity towards cisplatin-resistant ovarian cancer cells and it is promising for further investigation as anti-cancer agents.

## 1. Introduction

Cancer, among several diseases, is one of the most feared and challenging illnesses, consisting of several types that share a common feature: the disorderly growth of cells [1]. Chemotherapy uses drugs able to destroy the tumour cells and prevent their spreading. The *cis*-diamminedichloridoplatinum(II), *cis*-[PtCl_2_(NH_3_)_2_], known as cisplatin, is a drug widely used in chemotherapy for cancer treatment [2,3]. After the introduction of cisplatin in chemotherapy, a series of Pt-based analogous were developed, and carboplatin and oxaliplatin were also accepted as drugs to treat cancer. Currently, many Pt-based drugs have been used to treat colorectal, ovarian, lung and bladder cancer [4]. Despite the great success of platinum-based complexes, side effects are frequently observed and reported by the patients, including nausea, vomiting and hair loss, among others. Additionally, resistance of tumour cells to platinum-based complexes has been observed [2]. Thus, to minimize or eliminate these side effects, many efforts have been made to develop new metallodrugs that are more efficient and less harmful, or that have no side effects [5].

Hence, researchers are confronted with the challenge of developing new compounds that exhibit substantial antitumour activity, high selectivity and reduced toxicity in comparison to the drugs currently used in chemotherapy. In this sense, ruthenium complexes have been a promising alternative owing to their biological properties [6,7]. A widely used strategy in the research of novel compounds with biological activity involves coordinating ligands that possess inherent biological activity to a metal centre with the aim of obtaining new compounds with more significant biological activity owing to the synergistic effect that may occur [8,9]. Here, in this report, we have highlighted the class of natural products that include curcumin and its derivatives, as ligands [10].

Curcumin exhibits several medicinal properties [11] such as anti-inflammatory [12], antioxidant [13], antibacterial [14], anti-Alzheimer [15,16] and anti-cancer [17,18]. Regarding antitumour properties, curcumin has been studied against different types of cancer cells, such as breast [19], lung [20], ovarian [21] and prostate [17]. Metal complexes of curcumin and its derivatives are found in the literature with many d-block metals, including Ru, Fe, Os and Pt [22–24]. A series of ‘half-sandwich’ ruthenium/arene complexes containing curcumin-based ligands (curH = curcumin, bdcurH = bisdemethoxycurcumine) and PTA (1,3,5-triaza-7-phosphadamantane), with the general formulas [Ru(cym)(cur)-(PTA)][SO_3_CF_3_], [Ru(hmb)(cur)(PTA)]-[SO_3_CF_3_] and [Ru(hmb)(bdcur)(PTA)][SO_3_CF_3_], were active against human ovarian carcinoma cells (A2780 and A2780*cis*R) [25]. Also, other organometallic class, (p-cymene)Ru(curcuminoid)chloride, shows cytotoxicity against five tumour cell lines, being more active for the colorectal tumour HCT116, MCF-7 and A2780 cell lines; meanwhile, less sensitive against human glioblastoma U-87 and lung carcinoma A549 [26]. Recently, the antiproliferative activity of ruthenium diiminic complexes with curcuminoid against A2780 was evaluated and the complexes present ability to modulate the NF-κB transcription factor [27]. Therefore, considering the great interest in this ligand class, in the present report, the β-diketone derivatives [acetylacetonate (acac), dibenzoylmethanate (dbm), and curcuminate (cur)] were used as ligands to form three new complexes. The inspiration for the design of the new complexes came from the interesting results already observed by our research group, where the introduction of phosphine ligands substantially increased the cytotoxic activity of the complexes, which could be associated with increased hydrophobicity and enhanced cellular uptake [28–30]. Consequently, in addition to introducing the β-diketones, triphenylphosphine ligands were selected for developing the new complexes. The compounds were characterized by several physical techniques; their cytotoxic activity and interaction with *calf thymus* DNA (CT-DNA) were evaluated. This approach may be a good strategy for the development of new compounds with antitumour activity, and the results may be of interest for the inorganic-medicinal field.

## 2. Material and methods

### 2.1. Materials and physical measurement

All reactions were performed under argon atmosphere. The reagents and chemicals were of analytical grade and were used without purification. The solvents used in the development of this work were dichloromethane, methanol, toluene and ethyl ether from Synth or Merck, and they were used without prior treatment. Ruthenium trichloride trihydrate, triphenylphosphine (PPh_3_), 2,2′-bipyridine (bipy), ammonium hexafluorophosphate (NH_4_PF_6_), triethylamine (Et_3_N), curcumin (cur), dibenzoylmethane (dbm) and acetylacetonate (acac), from Aldrich, were used as supplied. The nuclear magnetic resonance (NMR) experiments (^31^P{^1^H}, ^1^H and ^13^C{^1^H}) were recorded on a Bruker Ascend 400 spectrometer of 5 mm inner diameter indirect probe with automatic tuning matching (ATM). Elemental analyses were conducted on a Fisons EA 1108 model equipment (Thermo Scientific). UV–Vis spectra were recorded using a Genesys 10S UV–Vis spectrophotometer (Thermo Scientific). The IR spectra were obtained using a Perkin Elmer FTIR (Fourier-transform infrared spectroscopy) spectrometer with the Attenuated Total Reflectance (ATR) method. Electrochemical assays were carried out by using a three-electrode system consisting of an Ag/AgCl (3.0 mol l^−1^ KCl) reference electrode and Pt disks as working and auxiliary electrodes. An Autolab Potentiostat/Galvanostate electrochemical analyser, model PGSTAT302N, was used to perform the electrochemical measurements, at room temperature, in CH_2_Cl_2_ containing 0.10 M tetrabutylammonium perchlorate (Fluka Purum) as a supporting electrolyte. The analysis was conducted by X-ray diffraction (XRD) on a Rigaku XtaLAB mini II diffractometer, using graphite monochromated MoKα (0.71073 Å) radiation.

### 2.2. General procedure to obtain ruthenium complexes containing β-dicetone

The precursor complex *cis*-[RuCl_2_(PPh_3_)_2_(bipy)] was obtained according to reported procedures described by Batista and coworkers [31]. First, in a Schlenk-type flask containing methanol (7.5 ml) and dichloromethane (15 ml) 1 : 2 (v/v), the *cis*-[RuCl_2_(PPh_3_)_2_(bipy)] (0.52 mmol, 500 mg) precursor complex was added. Then, the corresponding β-diketone (curH, acacH and dbmH) (0.23 mmol), 50 μl of triethylamine (Et_3_N) and NH_4_PF_6_ (9.5 mg) were added to the system. The reaction was kept under argon atmosphere and reflux for 18 h. Subsequently, the volume of the solution was reduced to approximately 1 ml, and a dark solid was precipitated by the addition of distilled water. The precipitate was filtered off, washed with distilled water, diethyl ether and dried under vacuum.

*[Ru(acac)(PPh*_*3*_*)*_*2*_*(bipy)]PF*_*6*_ *(**Ru1**)*: yield: (68%). Elemental analysis (%) for (RuC_51_H_45_F_6_N_2_O_2_P_3_): calculated C, 59.71; H, 4.42; N, 2.73; found: C, 59.18; H, 4.36; N, 2.67. Infrared (cm^−1^): 1564, 1522, 1483, 1431, 1398, 1275, 1185, 1090, 1025, 999, 831, 742, 697, 627, 555, 516. UV–Vis (dimethylsulphoxide (DMSO), 1.0 × 10^−4^ M) *λ*/nm (*ε*/M^−1^ cm^−1^): 275 (31 700), 334 (7700), 453 (3440). ^1^H NMR (400 MHz, acetone-d_6_) *δ* 9.61 (d, *J* = 5.6 Hz, 2H), 7.71–7.59 (m, 4H), 7.46 (td, *J* = 6.6; 2.3 Hz, 2H), 7.38 (tt, *J* = 7.4 Hz, 3H), 7.24 (tt, *J* = 7.6; 1.4 Hz, 12H), 7.14–7.05 (m, 12H), 4.71 (s, 1H), 2.88 (s, 3H), 1.55 (s, 6H). NMR of ^13^C{^1^H} (100 MHz, acetone-d_6_) *δ* 205.33 (Ru-O = C); 186.59 (Ru-O = C); 159.27; 150.83; 134.66; 133.45; 130.23; 129.71; 128.15; 124.95; 123.24; 101.07; 28.97. ^31^P{^1^H} ^1^H NMR (acetone-d_6_, 400 MHz) *δ* (ppm): 28.34 (P1, P2), −143.4 (hept, J_P−P_ *=* 19.7 Hz, PF_6_^−^).

*[Ru(dbm)(PPh*_*3*_*)*_*2*_*(bipy)]PF*_*6*_ *(**Ru2**)*: yield (72%). Elemental analysis (%) for (RuC_61_H_49_F_6_N_2_O_2_P_3_): calculated: C, 63.71; H, 4.29; N, 2.44; found: C, 64.20; H, 4.39; N, 2.34. Infrared (cm^−1^): 1589, 1520, 1481, 1450, 1436, 1389, 1303, 1225, 1186, 1090, 1025, 832, 742, 691, 554, 509. UV–Vis (DMSO, 1.0 × 10^−4^M) λ/nm (ε/M^−1^ cm^−1^): 296 (27 300), 340 (14 000), 425 (66 500). ^1^H NMR (400 MHz, acetone-d_6_) *δ* 9.75 (dd, *J* = 5.7; 1.4 Hz, 2H), 8.01–7.91 (m, 4H), 7.78 (dd, *J* = 8.1; 1.5 Hz, 2H), 7.72 (td, *J* = 7.7; 1.4 Hz, 2H), 7.61–7.53 (m, 2H), 7.54–7.44 (m, 6H), 7.25–7.14 (m, 5H), 7.08 (dtd, *J* = 8.3; 4.6; 1.5 Hz, 12H), 7.02 (ddt, *J* = 8.3; 7.0; 1.5 Hz, 12H). ^13^C{^1^H} NMR (100 MHz, acetone-d_6_) *δ* 205.32 (Ru-C=O); 180.65 (Ru-C=O); 159.56; 151.58; 138.88; 135.03; 133.40; 133.35; 133.30; 130.38; 129.86; 129.68; 129.58; 129.50; 128.16; 128.12; 128.07; 128.00; 127.05; 125.23; 123.37; 95.46. ^31^P{^1^H} NMR (acetone-d_6_, 400 MHz) *δ* (ppm): 28.54 (P1, P2), −143.5 (hept, *J*_P−P_ = 19.7 Hz, PF_6_^−^).

*[Ru(cur)(PPh*_*3*_*)*_*2*_*(bipy)]PF*_*6*_ *(****Ru3****)*: yield (83%). Elemental analysis (%) for (RuC_67_H_57_F_6_N_2_O_6_P_3_): calculated: C, 62.18; H, 4.44; N, 2.18; found: C, 62.03; H, 4.40; N, 2.07. Infrared (cm^−1^): 1625, 1599, 1503, 1425, 1265, 1163, 1122, 1091, 1026, 970, 831, 744, 692, 610, 556, 516. UV–Vis (DMSO, 1.0 × 10^−4^ M) *λ*/nm (ε/M^−1^ cm^−1^): 295 (38 800), 406 (shoulder), 425 (47 000). ^1^H NMR (400 MHz, acetone-d_6_) *δ* 9.73 (d, *J* = 5.7 Hz, 2H), 7.82–7.60 (m, 7H), 7.45 (ddd, *J* = 6.7; 5.6; 2.1 Hz, 2H), 7.38–7.22 (m, 12H), 7.13 (dq, *J* = 4.0; 1.9 Hz, 24H), 6.99 (d, *J* = 7.9 Hz, 2H), 6.26 (d, *J* = 15.7 Hz, 2H), 5.07 (s, 1H), 3.97 (s, 6H). ^13^C{^1^H} NMR (100 MHz, acetone-d_6_) *δ* 205.33 (Ru-C=O); 177.99 (Ru-C=O); 159.69; 151.20; 148.32; 148;02; 137.89; 134.63; 133.63; 133.58; 133.53; 130.04; 129.86; 129.68; 129.55; 128.65; 128.16; 125.83; 124.99; 123.25; 122.79; 115.94; 115.36; 109.31; 106.69; 55.46; 28.96. ^31^P{^1^H} NMR (acetone-d_6_, 400 MHz) *δ* (ppm): 27.01 (P1, P2), 143.4 (hept, *J*_P−P_ = 19.7 Hz, PF_6_^−^).

### 2.3. Crystal structure determination by X-ray diffraction

The molecular structures of **Ru1** and **Ru2** complexes were elucidated by single-crystal X-ray diffraction analysis. The crystals of complexes were obtained by slow diffusion using CH_2_Cl_2_ as the solvent and ethyl ether as the external solvent. Ru1 and Ru2 crystallize in a monoclinic system and to the space group P2_1_/c and P2_1_/n, respectively. The X-ray diffraction data were collected on a Rigaku XtaLAB mini II diffractometer using monochromated MoKα (0.71073 Å) radiation by graphite. The collected diffraction data were treated by specific crystallographic programmes. The WINGX program package was used in the data analysis. The structures were solved using direct methods with the SHELXS-97 program. The models, thus obtained, were refined (full matrix least squares) in F^2^ using the SHELXS-97 program. The program Mercury 4.0 was used to analyse and elaborate graphical representations of the structures. Crystallographic data were deposited at the Cambridge Crystallographic Data Centre as a supplementary publication with CCDC 2335556 for **Ru1** and CCDC 2335557 for **Ru2**. The copies of the data can be obtained free of charge via www.ccdc.cam.ac.uk/conts/retrieving.html (or from the Cambridge Crystallographic Data Centre, CCDC, 12 Union Road, Cambridge, CB2 1EZ, UK; fax:+44-1223-336033; or e-mail: deposit@ccdc.cam.ac.uk).

### 2.4. Cell culture and MTT (3-[4,5-dimethylthiazol-2-yl]-2,5 diphenyl tetrazolium bromide) assay

The cytotoxic activity of complexes (**Ru1**–**Ru3**), of the free ligands acac, dmb and cur, as well as cisplatin, were studied against MDA-MB-231 (triple-negative breast cancer, ATCC: HTB-26), MCF-7 (hormone-dependent breast cancer, ATCC: HTB−22), A549 (lung cancer, ATCC: CCL-185) and A2780*cis* (cisplatin-resistant ovarian cancer), and the non-tumour cell lines: MCF-10A (breast epithelial cells, ATCC: CRL-10317) and MRC-5 (lung fibroblast, ATCC: CCL-171). The MDA-MB-231, A549 and MRC5 cell lines were cultured in Dulbecco’s modified eagle medium (DMEM) containing 10% fetal bovine serum (FBS), penicillin (100 IU ml^−1^), streptomycin (100 mg ml^−1^) and l-glutamine (2 mM). The MCF-7 and A2780*cis* cells were grown in the Roswell Park Memorial Institute (RPMI) containing 10% FBS, penicillin (100 IU ml^−1^), streptomycin (100 mg ml^−1^) and l-glutamine (2 mM). The MCF-10A cells were grown in DMEM nutrient mixture F-12 (DMEM F-12) containing 5% fetal horse serum, epidermal growth factor (EGF) (20 ng ml^−1^), hydrocortisone (0.5 µg ml^−1^), insulin (0.01 mg ml^−1^), 1% penicillin and 1% streptomycin. All the cells were maintained at 37°C in a humidified incubator with a 5% CO_2_ atmosphere.

The cytotoxic activities of the free ligands and complexes were analysed by MTT (3-[4,5-dimethylthiazol-2-yl]-2,5 diphenyl tetrazolium bromide) assay [32]. For that, cells were plated in 96-well plates (1.5 × 10^4^ cells well^−1^) and kept for 24 h in an incubator with 5% CO_2_ and 37°C for adherence. Then, the cells were treated with the compounds, solubilized in DMSO, at different concentrations and the plates were incubated for 48 h. To solubilize cisplatin, dimethylformamide (DMF) was used. The percentage of DMSO and DMF used in the assay was 0.5%. Afterwards, 50 μl of MTT (0.5 mg ml^−1^ in phosphate-buffered saline) was added to each well of the plate and the plates were incubated for 4 h at 37°C. Then, the culture medium was removed, and the water-insoluble formazan crystals formed were solubilized by the addition of 100 μl isopropanol. The absorbance was recorded at 540 nm using a microplate reader. The half maximal inhibitory concentration (IC_50_) values were obtained using the GraphPad Prism software.

### 2.5. DNA interaction studies by spectroscopic titration

Initially, a DNA stock solution was prepared by adding 2 mg of CT-DNA in 1 ml of Tris-HCl buffer (4.5 mmol l^−1^ Tris- HCl, 0.5 mmol l^−1^ Tris-base and 50 mmol l^−1^ NaCl) at a pH of 7.4. The concentration of CT-DNA was determined by UV–Vis. In a cuvette containing Tris-HCl buffer (2000 µl), 80 µl of the CT-DNA stock solution was added, and the UV–Vis spectrum was recorded. The concentration of the CT-DNA stock solution prepared (3.66 mmol l^−1^) was determined from the absorbance at 260 nm, using molar absorptivity of CT-DNA (6600 mol^−1^ cm^−1^ l), and the optical path (*b* = 1 cm), employing the Lambert–Beer law: *A*_260_ = *ε*_260_ × *b* × *C*.

The spectroscopic titrations were performed by employing two cuvettes: to cuvette 1, 1200 μl of DMSO and 1800 μl of Tris-HCl buffer were added, corresponding to the blank measurements; and to cuvette 2, 1800 μl of Tris-HCl buffer and 200 μl of the stock solution from the complex in DMSO were added, totalling a concentration of 1.82 × 10^−2^ mmol l^−1^ of the complex in the cuvette (concentration at which the absorbance is approximately 1). Then, successive additions of 10 μl aliquots of CT-DNA at 3.66 mmol l^−1^ were made in both cuvettes. For each addition of DNA, the solutions were homogenized for 1 min to reach the equilibrium condition, and then the UV–Vis spectrum was recorded. The procedure was performed in triplicate for the three synthesized complexes and from the data obtained it was possible to determine the binding constant (*K*_b_) between the complexes and CT-DNA. The binding constants between complex and CT-DNA were determined from the graph [DNA]/(ε_a_ − ε_f_) versus [DNA] and the following equation [33]:


$$\frac{\text{[DNA]}}{\text{(}\epsilon_{a}\text{-}\epsilon_{f}\text{)}}\text{=}\frac{\text{[DNA]}}{\text{(}\epsilon_{b}\text{-}\epsilon_{f}\text{)}}\text{+}\frac{\text{1}}{\text{[}K_{b}\left(\epsilon_{b}-\epsilon_{f}\right)\text{]}},$$

where *ε*_a_ is the apparent extinction coefficient, which corresponds to the ratio between the measured absorbance and the concentration of the complex ((A*_observed_*/complex]); *ε*_f_ corresponds to the molar absorptivity of the free complex (without DNA addition); *ε*_b_ is the molar absorptivity of the complex bound to DNA and *K*_b_ corresponds to the binding constant.

## 3. Results and discussion

### 3.1. Synthesis and characterization

**Figure 1.**
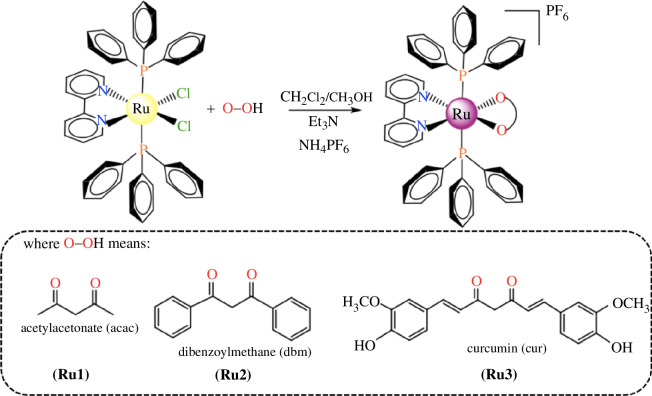
Synthetic route used to obtain the Ru(II) complexes with *β*-diketones ligands.

Three new complexes with general formula [Ru(O-O)(PPh_3_)_2_(bipy)]PF_6_ were obtained from the reaction of *cis*-[RuCl_2_(PPh_3_)_2_(bipy)] precursor with the β-diketones acetylacetonate (acac) (**Ru1**), dibenzoylmethanate (dbm) (**Ru2**) and curcuminate (cur) (**Ru3**) (figure 1).

The cationic complexes [Ru(acac)(PPh_3_)_2_(bipy)]^+^ (**Ru1**), [Ru(dbm)(PPh_3_)_2_(bipy)]^+^ (**Ru2**) and [Ru(cur)(PPh_3_)_2_(bipy)]^+^ (**Ru3**) were obtained in 68, 72 and 83% of yields, respectively, containing PF_6_^−^ as anion. The purity and structures of complexes were confirmed by several techniques in the solid state and in solution.

Free ligands present absorption bands at 3293 and 3508 cm^−1^, indicating the presence of the *ν*(O–H) stretching vibration. A strong absorption band at 1625 cm^−1^ has a predominant character of the stretching and *ν*(C=C). Also, another band at 1602 cm^−1^ corresponds to the vibrational modes of *ν*(C=C) stretching of aromatic rings and at 1502 cm^−1^ is assigned to *ν*(C=O). Finally, the *ν*(C-O) and *ν*(C-O-C) stretch vibrations are observed at 1271 cm^−1^ and at 1026 cm^−1^, respectively. Each one of the complexes **Ru1**, **Ru2** and **Ru3** does not exhibit a broad band between 3400 and 2600 cm^−1^, indicating a ligand deprotonation, allowing metal coordination [34]. Also, the absorption band corresponding to the *ν*(C=O) stretch is shifted to lower frequency, located between 1599 and 1520 cm^−1^ after metal coordination. Similarly, in all cases, the absorption bands between 1490 and 1425 cm^−1^ can be attributed to the ν(C-H) stretching of the 2,2′-bipyridine ring deformation upon complexation with the Ru metal. In addition to the absorption band at around 520 cm^−1^ corresponding to *ν*(Ru-O) [35,36], the bands around 556 and 831 cm^−1^ can be attributed to P-F stretching vibration from the PF_6_^−^ counterion [36].

The ^1^H NMR spectra of the ruthenium complexes showed a signal at around 9.7 ppm corresponding to the orthobipyridine hydrogen atoms. Integration data confirm the presence of 45 hydrogen atoms for the **Ru1** complex, 49 for **Ru2** and 57 for **Ru3**, as expected for each case. Similarly, the ^1^H NMR spectrum of **Ru1** shows two singlets, one at 1.55 ppm corresponding to the methyl groups with a relative integral of 6H and another at 4.70 ppm with a relative integral of ^1^H corresponding to the ^1^H*α*-dicarbonyl. In the region of the aromatic hydrogens, the signals are between 7.68 and 7.63 ppm, with a relative integral of 4H, a duplet triplet at 7.48–7.45 ppm with a relative integral for 2H (J = 6.4 and 2.2 Hz), a triplet at 7.40–7.36 (*J* = 7.40 Hz) with a relative integral for 6H, a triplet at 7.24 ppm (*J* = 7.70 Hz) for 12H and a multiplet at 7.10 ppm with relative integral for 12H.

The ^13^C{^1^H} NMR spectra of **Ru1–Ru3** complexes showed signals corresponding to the carbonyl group at 205.3 ppm and 181.7 ppm, characteristic of the enolic tautomer (C=CO) after metal coordination. For complex Ru1, this fragment corresponds to chemical shifts at 205.3 ppm (C=O) and 186.6 ppm (C–O), respectively. Similar signals are observed at 205.3 and 180.6 ppm for **Ru2** and for **Ru3** at 205.3 and 178.0 ppm.

The ^31^P{^1^H} NMR spectrum of the *cis,trans*-[RuCl_2_(PPh_3_)_2_(bipy)] exhibits a simplet with a chemical shift at approximately *δ* = 20.0 ppm, owing to the chemical equivalence of the phosphorus atoms that are positioned *trans* to each other. Furthermore, during the analysis of the ^31^P{^1^H} NMR spectrum of the **Ru3** complex, a simplet is observed at *δ* = 26.3 ppm thus indicating the chemical equivalence of the phosphine ligands (PPh_3_). However, the shift of the phosphorus signal towards higher frequencies compared with the precursor is a result of curcumin coordination through the oxygen atoms, which deshields the phosphorus atoms more than the chlorido bonds (characterized by σ- and π-donors). Similarly, for complexes Ru1 and Ru2, their ^31^P{^1^H} NMR spectra show simplets at *δ* = 28.3 and at *δ* = 28.5 ppm, respectively, relating to the magnetic equivalence of the two PPh_3_ ligands *trans* to each other.

The UV–Vis absorption spectra of the **Ru1–Ru3** complexes, obtained in DMSO, have been provided in the electronic supplementary material. The UV–Vis spectra of the free ligands (acac, cur and dbm) show bands of π→π∗, n→π∗ transitions in the wavelength range of 274–433 nm. However, for complexes **Ru1–Ru3**, the UV–Vis spectra display bands associated with metal–ligand charge transfer complexes. These bands arise from the transitions of the 4d^6^ orbitals of Ru(II) to the π∗ orbitals of the ligands (4d^6^ Ru(II)→π∗), and they are observed within the range between 334 and 425 nm. Additionally, bands present in the region of 275–296 nm are attributed to intraligand π → π∗ transitions.

### 3.2. Electrochemical studies

Table 1 presents the results of the differential pulse and cyclic voltammetry experiments conducted on the **Ru1–Ru3** complexes in dichloromethane. Notably, **Ru1** and **Ru2** complexes demonstrated reversible processes (*I*_pa_/*I*_pc_ ≈ 1) that can be attributed to the oxidation processes of Ru(II)/Ru(III). The complexes **Ru1** (*E*_pa_ = 0.98 V), **Ru2** (*E*_pa_ = 1.03 V) and **Ru3** (*E*_pa_ = 0.92 V) exhibit a notable shift in oxidation potentials towards more positive values when compared with the precursor (*E*_pa_ = 0.54 V). This shift can be attributed to the replacement of two σ- and π-donor chloride ligands with the O–O donor ligands. This aspect indicates a higher stability of the metal centre upon coordination of the negatively charged monoanionic ligand. Additionally, the pronounced electronegativity of the oxygen atoms reduces the σ-donating ability of the O–O region, and consequently, decreases the electronic density around the ruthenium centre. As a result, the oxidation potential increases.

**Table 1.** Electrochemical parameters obtained by cyclic voltammetry.

complexes	*E*_pa_ (V)	*E*_pc_ (V)	*E*_1/2_ (V)	*I*_pa_/*I*_pc_
*cis*-[RuCl_2_(PPh_3_)_2_(bipy)]	0.54	0.38	0.46	0.78
Ru1	0.98	0.82	0.90	0.93
Ru2	1.03	0.87	0.95	0.93
Ru3	0.92	0.78	0.85	1.19

### 3.3. Crystal structure of Ru1 and Ru2 complexes

**Figure 2.**
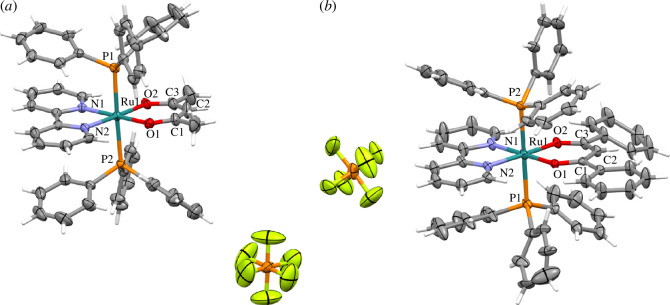
The crystal structure of (*a*) Ru1 and (*b*) Ru2 complexes with the selected atoms labelled and the ellipsoids with 30% probability.

**Ru1** and **Ru2** crystals were obtained by slow vapour diffusion, using CH_2_Cl_2_ as a solvent and ethyl ether as an external solvent. **Ru1** and **Ru2** complexes crystallize in a monoclinic system and to the space groups P2_1_/c and P2_1_/n, respectively. The ruthenium coordinates to two oxygen atoms of the β-dikethonate ligand, with similar distances (Ru1-O2) of 2.062 Å and (Ru1-O1) 2.063 Å, for complex **Ru1** and **Ru2** with similar distances (Ru1-O2) of 2.056 Å and (Ru1-O1) 2.060 Å. Additionally, in complex **Ru1**, the bond distances between C1-O1 and C3-O2 are 1.267 Å and 1.261 Å, respectively. The difference in the bond lengths between C1–C2 (1.293 Å) and C2–C3 (1.389 Å) indicates a higher π character between C1 and C2, corresponding to the enol fragment of the ligand. The crystal structures are depicted in figure 2.

### 3.4. The interaction of complexes with *calf thymus* DNA by spectroscopic titration

**Figure 3.**
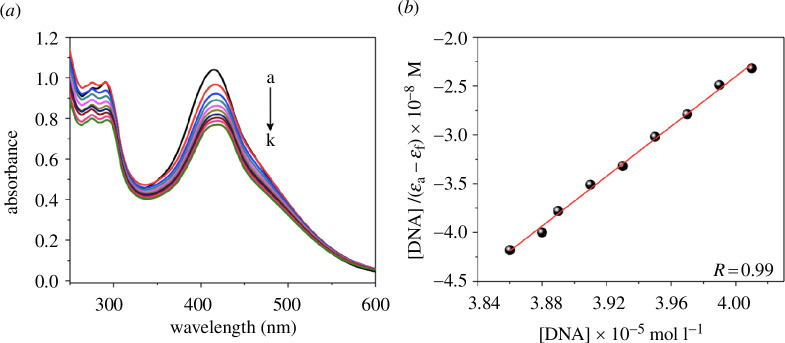
(*a*) Spectrophotometric titration spectra of Ru3 complex (1.5 × 10^−4^ mol l^−1^) with CT-DNA at different concentrations. (*b*) Plot of [DNA]/(*ε*_a_ − *ε*_f_) versus [DNA] for the titration, to obtain *K*_b_ constant.

The interaction of the synthesized complexes with DNA was investigated using spectroscopic titration in the UV–Vis region. By measuring the changes in electronic absorption in the UV–Vis region, the interaction between DNA and small molecules can be easily studied. In order to examine the interaction, the UV–Vis electronic spectra of **Ru1**, **Ru2** and **Ru3** complexes were collected both in the absence and presence of CT-DNA. Figure 3 displays the spectrum for **Ru3** as an example. The initial spectrum (black curve) corresponds to the solution of the complex without CT-DNA, while the subsequent curves were obtained after successive additions of CT-DNA to the complex solution.

As shown in figure 3, the addition of CT-DNA to the solution containing the complex induces a decrease in absorbance, a phenomenon known as hypochromism. This decrease in absorbance is attributed to the interaction of the **Ru3** complex with CT-DNA. As small amounts of CT-DNA are incrementally added, no significant changes in the *λ*_max_ values are observed. The interaction facilitates coupling between the π∗ orbital of the complex and the π orbital of the nitrogenous base pairs present in DNA. As a result, there is partial filling of these orbitals by electrons, leading to a decrease in transition energy for the π→π∗ transition, causing hypochromism.

The values of the binding constants between the complexes and CT-DNA (*K*_b_) were determined, and the magnitudes were (0.31 ± 0.01) × 10^3^, (1.54 ± 0.12) × 10^4^ and (4.25 ± 0.75) × 10^3^ l mol^−1^, for **Ru1**, **Ru2** and **Ru3** complexes, respectively. The *K*_b_ values obtained for the synthesized complexes are comparatively lower than the constants determined for ruthenium complexes tested under similar conditions [37,38]. Also, the values are lower than the *K*_b_ value of a classic DNA intercalator such as ethidium bromide (*K*_b_ = 10^6^ l mol^−1^) [39]. The results also suggest a weak interaction of **Ru1–Ru3** complexes with CT-DNA, possibly of an electrostatic nature, indicating that the complexes may cause less damage to the DNA.

### 3.5. Cytotoxic activity of Ru1–Ru3 complexes

The cytotoxicity of the **Ru1–Ru3** complexes, the free ligands (cur, acac, dbm) and the reference metallodrug cisplatin was evaluated using the MTT colourimetric assay. The obtained IC_50_ values (lowest concentration of the complex inhibiting 50% of cell growth) were used to compare the cytotoxic activity of each compound against different cell lines. The cell lines tested included MDA-MB-231 (triple-negative breast cancer), MCF-7 (hormone-dependent breast cancer), A549 (lung cancer) and A2780*cis* (cisplatin-resistant ovarian cancer), as well as non-tumour cell lines MCF-10A (breast epithelial cells) and MRC-5 (lung fibroblast). In order to assess the cytotoxicity compared with cisplatin, a reference metallodrug, the IC_50_ values and selectivity index (SI) were calculated and are provided in tables 2 and 3.

**Table 2.** The cytotoxicity activity (IC_50_) of ruthenium complexes, free ligands and cisplatin, against MDA-MB-231, MCF-7 and MCF-10A cell lines, respectively. (SI^1^ = IC_50_ (MCF-10A)/IC_50_ (MDA-MB-231) and SI^2^ = IC_50_ (MCF-10A)/IC_50_ (MCF-7).)

	IC_50_ (μM)				
compounds	MDA-MB-231	MCF-7	MCF-10A	SI^1^	SI^2^
Ru1	2.09 ± 0.46)	(1.86 ± 0.20)	(1.70 ± 0.03)	0.81	0.91
Ru2	(3.37 ± 0.33)	(1.74 ± 0.23)	(1.71 ± 0.23)	0.50	0.98
Ru3	(6.80 ± 1.74)	(13.98 ± 2.02)	(6.41 ± 0.88)	0.94	0.45
cur	(29.69 ± 0.95)	(19.98 ± 2.25)	(16.88 ± 0.76)	0.57	0.84
dbm	(59.65 ± 1.42)	(11.68 ± 1.14)	(50.68 ± 1.40)	0.85	4.34
acac	>100	>100	>100	–	–
cisplatin	(10.2 ± 0.2)	(13.98 ± 2.02)	(29.45 ± 0.85)	2.88	2.10

**Table 3.** Cytotoxicity activity (IC_50_) of ruthenium complexes, free ligands and cisplatin, against A2780*cis*, A549 and MRC-5 cell lines, respectively. (SI^3^ = IC_50_ (MRC-5)/IC_50_ (A2780*cis*) and SI^4^ = IC_50_ (MRC-5)/IC_50_ (A549).)

	IC_50_ (μM)					
compounds	A2780*cis*		A549	MRC-5	IS^3^	IS^4^
Ru1	(0.59 ± 0.06)		(1.43 ± 0.35)	(4.92 ± 0.39)	8.34	3.44
Ru2	(0.39 ± 0.09)		(1.06 ± 0.24)	(4.85 ± 0.50)	12.43	4.57
Ru3	(0.82 ± 0.01)		>30	>30	–	–
cur	(4.02 ± 0.36)		(33.33 ± 0.36)	(8.99 ± 0.63)	2.24	0.27
dbm	(62.63 ± 5.88)		(23.12 ± 3.40)	(9.54 ± 0.73)	0.15	0.41
acac	>100		>100	>100	–	–
cisplatin	(37.03 ± 5.11)		(11.54 ± 1.19)	(29.09 ± 0.78)	0.78	2.52

The free ligand acac did not exhibit activity under the tested concentrations (<100 µM), and cur and dbm exhibit cytotoxic activity against the cell lines investigated. However, the cytotoxic activity of the complexes against tumour cell lines was significantly higher than that of their respective free ligands. The coordination of the ligands resulted in the formation of new complexes with substantially increased activity compared with the free ligands.

The complexes displayed cytotoxic activity against MCF-7, MDA-MB-231, A2780*cis* and A549 tumour cell lines, except for complex (3) against MCF-7 and A549 cell lines. Interestingly, the complexes exhibited their highest cytotoxic activity (lowest IC_50_ values) in ovarian and lung cell lines. In particular, the IC_50_ values of the complexes in ovarian cells ranged from 0.59 to 0.82 µM, while in MCF-7 breast tumour cells, the values were 1.86 to >30 µM.

**Ru1** and **Ru2** complexes demonstrated comparable cytotoxicity against the tumour cell lines. Notably, the complex containing the dbm ligand showed slightly higher cytotoxicity against the ovarian cancer strain A2780*cis* when compared with **Ru1** and **Ru3**. Conversely, **Ru3** exhibited the least cytotoxicity among all evaluated cell lines. **Ru1** and **Ru2** exhibit equal or greater cytotoxic activity compared with cisplatin against breast tumour cell lines (MDA-MB-231 and MCF-7). Specifically, in MCF-7 cells, **Ru1** and **Ru2** demonstrated IC_50_ values that were 7.5- and 8.0-fold lower than that of cisplatin, respectively.

By contrast, the **Ru1** complex, which contains the acac ligand, displayed three times higher toxicity than the **Ru3** complex with the cur ligand. It is worth noting that the **Ru3** complex, with the lowest cytotoxic activity among the synthesized complexes, features the bulkiest ligand. This suggests that the bulkiness of the ligand may influence cellular uptake, and consequently, impact cytotoxicity. Notably, all the synthesized complexes exhibited remarkable cytotoxicity, especially when compared with A2780*cis* cells, which are known to be resistant to cisplatin. Among the synthesized complexes, **Ru2** demonstrated the highest cytotoxicity. The cytotoxicity against A2780*cis* cells for **Ru1–Ru3** is comparable with Ruthenium(II) complexes containing the 1,3,5-triaza-7-phosphaadamantane (PTA) and curcumin-based ligands, showing the relevance of curcuminoid coordination to improve the activity against cisplatin-resistant cells.

Furthermore, in order to evaluate their potential as metallodrugs, the complexes’ SI was obtained. The SI indicates the ability of the complex to be more cytotoxic to tumour cells than non-tumour cells, calculated as the ratio of IC_50_ values between non-tumour and tumour cell lines. Based on the SI values, it is observed that the synthesized complexes with SI values below 1 are not selective for the MDA-MB-231 or MCF-7 breast tumour cells.

Regarding the A549 cells, both **Ru1** and **Ru2** were more cytotoxic than cisplatin. The IC_50_ value of cisplatin was 8- and 11-fold higher than the IC_50_ values of **Ru1** and **Ru2** complexes, respectively. Furthermore, the complexes exhibited greater selectivity for A549, the lung tumour cells, compared with the non-lung tumour cells, MRC-5, and they were even more selective than the cisplatin.

## 4. Conclusion

In this study, we presented the syntheses and characterizations of three novel complexes with the general formula [Ru(O-O)(PPh_3_)_2_(bipy)]PF_6_, where O–O represents β-diketones (acetylacetonate, dibenzoylmethane and curcumin), which are ligands of biological interest. The chemical structures of these new complexes were elucidated using various techniques. Notably, the new complexes exhibited significantly higher cytotoxic activity against various tumour cell lines compared with the free ligands. Additionally, **Ru1** and **Ru2** demonstrated higher selectivity when compared with the reference drug, cisplatin. Moreover, the **Ru1–Ru3** complexes revealed weak, possibly electrostatic interactions, with the CT-DNA. Our findings indicate the potential of these newly synthesized complexes as promising candidates for further investigation as cytotoxic agents, considering their enhanced activity against tumour cells and their favourable selectivity profile.

## Data Availability

The datasets correspond to the spectra collected for the structural characterization of the compounds reported in this article and are deposited as the electronic supplementary material [40]. Data are available from the Dryad Digital Repository [41].
